# Stroke and Pulmonary Thromboembolism Complicating a Kissing Aneurysm in the M1 Segment of the Right MCA

**DOI:** 10.3390/jcm14020564

**Published:** 2025-01-17

**Authors:** Corneliu Toader, Felix-Mircea Brehar, Mugurel Petrinel Radoi, Matei Serban, Razvan-Adrian Covache-Busuioc, Ghaith S. Aljboor, Radu Mircea Gorgan

**Affiliations:** 1 Department of Neurosurgery, “Carol Davila” University of Medicine and Pharmacy, 020021 Bucharest, Romania; corneliu.toader@umfcd.ro (C.T.); felix.brehar@umfcd.ro (F.-M.B.); razvan-adrian.covache-busuioc0720@stud.umfcd.ro (R.-A.C.-B.); ghaith-saleh-radi.aljboor@drd.umfcd.ro (G.S.A.); radugorgan@umfcd.ro (R.M.G.); 2 Department of Vascular Neurosurgery, National Institute of Neurology and Neurovascular Diseases, 077160 Bucharest, Romania; 3 Department of Neurosurgery, Clinical Emergency Hospital “Bagdasar-Arseni”, 041915 Bucharest, Romania; 4 Puls Med Association, 051885 Bucharest, Romania

**Keywords:** kissing aneurysm, microsurgical clipping, stroke, pulmonary thromboembolism, subarachnoid hemorrhage

## Abstract

**Background/Objectives:** Kissing aneurysms, a rare and intriguing cerebrovascular anomaly, challenge even the most advanced neurosurgical techniques. These lesions, characterized by two intimately apposed aneurysms with shared arterial walls, often masquerade as single, irregular aneurysms. This report documents a case of ruptured kissing aneurysms in the M1 segment of the right middle cerebral artery (MCA), complicated by ischemic stroke and pulmonary thromboembolism (PTE)—a convergence of severe complications rarely encountered. The case underscores the importance of precise diagnostics, innovative surgical strategies, and multidisciplinary care. **Methods:** A 55-year-old female presented with subarachnoid hemorrhage, confirmed by advanced imaging to arise from ruptured kissing aneurysms in the M1 segment of the right MCA. Surgical intervention via a right frontotemporal craniotomy and microsurgical clipping achieved definitive aneurysmal exclusion. Postoperatively, the patient experienced ischemic stroke and PTE, necessitating dynamic adjustments in anticoagulation therapy, intensive care, and rehabilitation protocols. **Results:** The dual aneurysms were successfully clipped, as confirmed by intraoperative and postoperative imaging. Despite developing significant complications, including left-sided motor deficits and PTE, a carefully orchestrated treatment strategy enabled the patient’s full recovery, with marked neurological and systemic improvement by her three-month follow-up. This favorable outcome highlights the resilience of a multidisciplinary approach in navigating such high-risk scenarios. **Conclusions:** This case showcases the formidable challenges of managing kissing aneurysms, particularly when compounded by stroke and PTE. It emphasizes the transformative role of cutting-edge imaging and surgical techniques in achieving successful outcomes. By illustrating how precision medicine and collaborative care can overcome rare and complex cases, this report contributes valuable insights to the evolving field of cerebrovascular surgery and postoperative management.

## 1. Introduction

The concept of aneurysmal “complexity” has undergone substantial evolution in recent years, largely attributable to the rapid advancements in endovascular therapies. Conditions previously deemed too challenging for conventional surgical clipping are now frequently managed with endovascular approaches, and conversely, some aneurysms once considered ideal for endovascular intervention may now necessitate microsurgical solutions [1]. Despite the ongoing progress in treatment techniques, the precise global prevalence of complex aneurysms remains poorly defined [2], and they continue to present formidable challenges for both neurosurgeons and interventional radiologists alike. The literature has delineated several criteria for defining aneurysmal complexity, including fusiform morphology, large or giant aneurysms, sac-originating vessels, thrombosis, previous interventions, wide-neck configurations, tortuous proximal vessels, and calcified segments [3].

Kissing aneurysms represent a rare and distinct category of complex aneurysms, characterized by two closely apposed aneurysms originating from separate arterial walls that share partially adherent structures [4]. Their anatomical proximity and irregular morphology often lead to misdiagnosis as single, lobulated aneurysms, necessitating advanced imaging techniques for accurate characterization. Within the broader framework of aneurysmal complexity, kissing aneurysms exhibit features such as wide-neck configurations, shared arterial walls, and close spatial alignment, which complicate both diagnosis and intervention. This is particularly relevant for cases involving the middle cerebral artery (MCA), where the vascular anatomy amplifies the challenges of surgical or endovascular approaches [5].

The Unruptured Intracranial Aneurysm Treatment Score (UIATS) characterizes aneurysmal complexity based on factors such as a neck diameter wider than the parent vessel, a calcified dome, tortuosity or stenosis of the proximal vessels, thrombosis within the aneurysm, or a sac size smaller than 3 mm [4,5]. Additionally, giant aneurysms and those that have previously undergone surgical or endovascular interventions are likewise categorized as complex [6,7,8]. The Berlin Classification, specifically addressing aneurysms of the MCA, underscores the critical role of these complexity factors in shaping best practices, particularly in cases involving thrombosed aneurysms [7]. The presence of any combination of these features can render conventional clipping techniques inadequate or unfeasible. Although recent definitions and classifications of aneurysmal complexity have emerged [1,3,4], they frequently lack a quantitative framework that integrates the qualitative attributes of these features and their implications for treatment strategies [9,10]. In a multidisciplinary context, especially concerning MCA aneurysms—among the most commonly surgically treated—this correlation between complexity features and treatment modalities is of paramount importance. Accurate preoperative identification of complexity markers is essential to anticipate and mitigate potential complications during surgical or endovascular intervention.

## 2. Case Report

A 55-year-old female patient was admitted to our clinic after being transferred from another hospital where she presented with severe headache, vertigo, and nonspecific balance disturbances, with a CT scan where subarachnoid hemorrhage was revealed in the right sylvian fissure, raising suspicion of an aneurysm at the bifurcation of the right MCA. Upon admission to our clinic, a neurological examination revealed moderate neck stiffness, signs of intracranial hypertension, and a positive Babinski sign bilaterally, symptoms associated with Hunt–Hess scale grade III.

**Figure 1:**
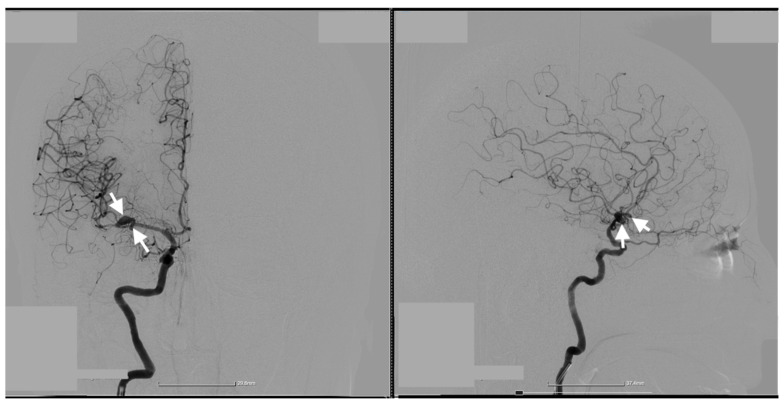
Preoperative two-dimensional (2D) digital subtraction angiography. Frontal (**left** image) and Profile (**right** image) 2D DSA shows the two kissing aneurysms located at the bifurcation of the M1 segment of the right MCA. The white arrows indicate the precise location of the aneurysms, highlighting their proximity and shared arterial walls.

**Figure 2:**
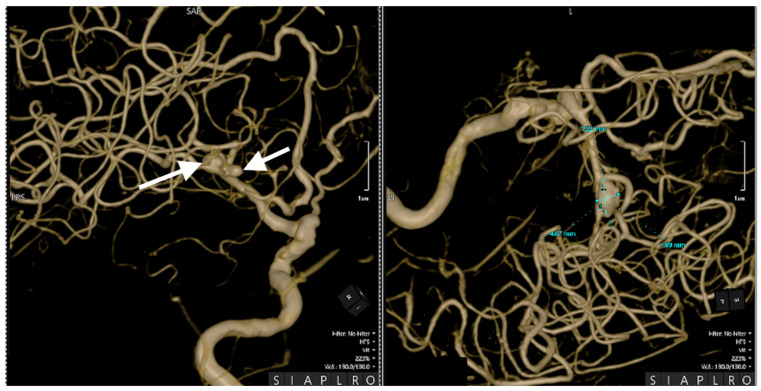
Preoperative three-dimensional (3D) DSA rotational angiography. The 3D reconstruction of rotational DSA (**left** image) highlights the two kissing aneurysms (white arrows), one with 3 mm diameter and the other with a 7 mm diameter (**right** image).

**Figure 3:**
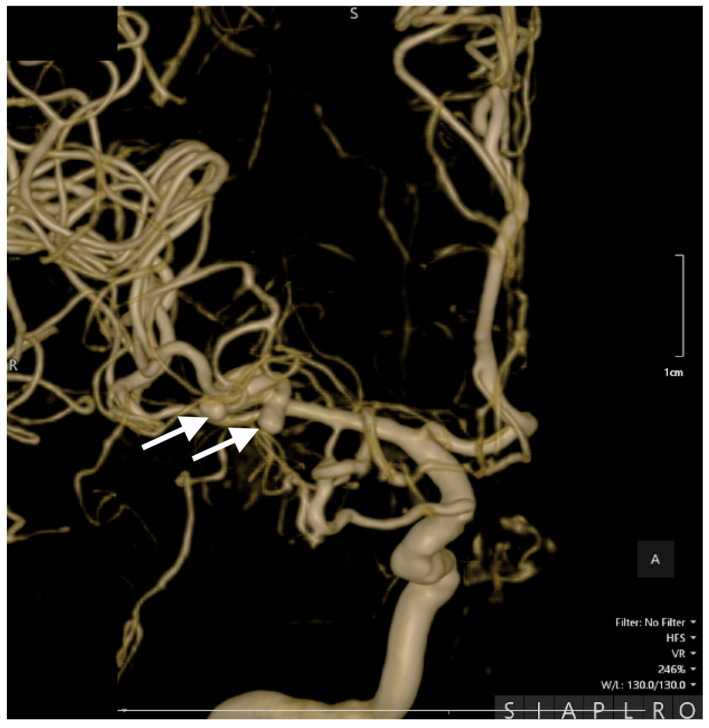
Preoperative 3D DSA rotational angiography. In a high-resolution image, two kissing aneurysms are seen on the bifurcation of the M1 segment of the right MCA. The white arrows indicate the aneurysms' distinct locations, emphasizing their close proximity and shared arterial structures, which are characteristic of this rare vascular anomaly.

Cerebral angiography confirmed the presence of 2 ruptured saccular aneurysms at the bifurcation of the M1 segment of the right MCA (Figure 1, Figure 2 and Figure 3).

During surgery, a right frontotemporal craniotomy was performed. The dura mater was incised, and under the operating microscope, the right sylvian cistern and the opto-carotid cistern were dissected to identify the bifurcation of the right internal carotid artery and the M1 segment of the right MCA. Two bifurcation aneurysms, described as “kissing aneurysms” with diameters of 3 mm and 7 mm, were identified. A temporary clip was applied to the M1 segment before the larger aneurysm was opened to allow the placement of a 5 mm Yasargil clip on its neck. Subsequently, a 3 mm Yasargil clip was applied to the smaller aneurysm. Hemostasis was achieved with electrocoagulation, Surgicel, and tamponade. The dura was sutured, the bone flap was replaced over an epidural drain, and the wound was closed in anatomical layers.

**Figure 4:**
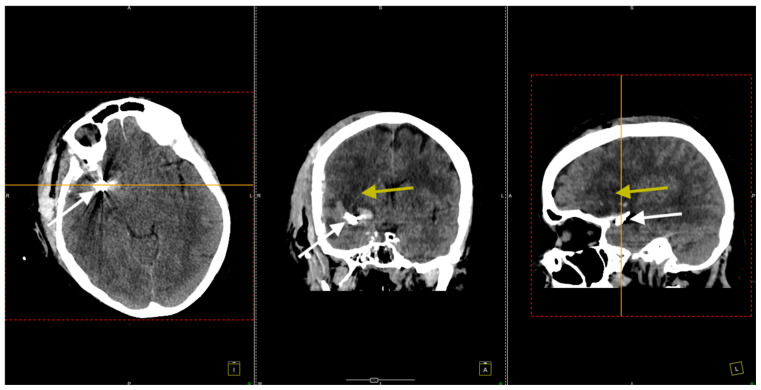
Postoperative CT scan. All three sections, transversal (**left** image), coronal (**middle** image), and sagittal (**right** image), show clip placement (white arrows) and sequelae from the ischemic stroke. The yellow arrows indicate areas of hypodensity corresponding to ischemic stroke sequelae in the right frontotemporal region.

Postoperative cerebral CT scans showed a right frontotemporal hypodensity suggestive of sequelae of the ischemic stroke, correlating with her left-sided motor deficit (Figure 4).

**Figure 5:**
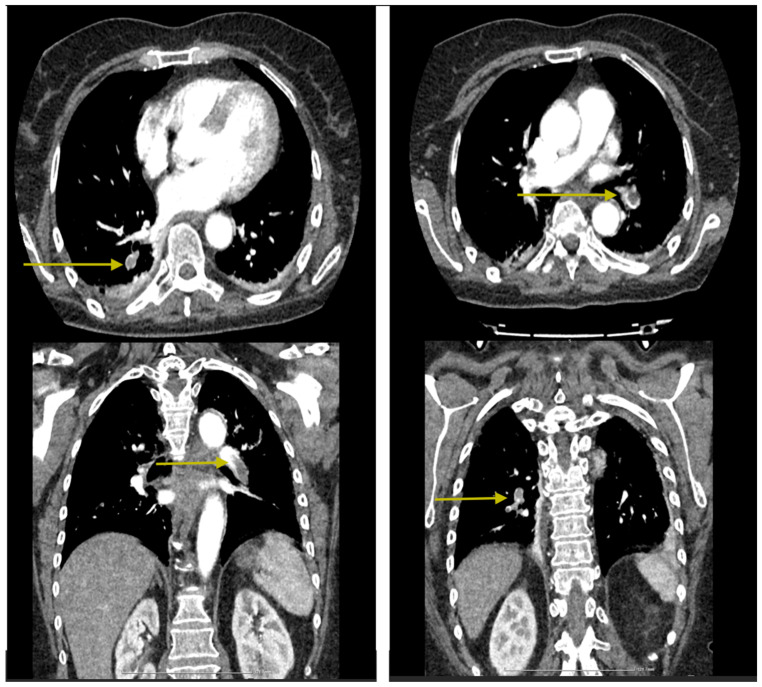
Intraluminal filling defects are observed in the pulmonary arteries supplying the basal pyramids and the medial segment of the middle lobe, with an appearance suggestive of pulmonary thromboembolism at this level (yellow arrows). Minimal passive subpleural atelectasis is noted bilaterally in the posterior-basal regions. No nodular expansive pulmonary lesions with a tumoral substrate are present. There are emphysematous bullae with a paraseptal apical distribution on the right side. No pleuro-pericardial fluid accumulations are observed. No mediastinal or axillary lymphadenopathy is detected bilaterally. The tracheobronchial tree is within normal limits. Intimal calcifications are present in the thoracic aorta.

Postoperatively, the patient developed a motor deficit, predominantly affecting the left arm. Her neurological status improved gradually, and she remained conscious, cooperative, and oriented in time and space. Two weeks after the surgical procedure, our patient experienced episodes of dyspnea due to pulmonary thromboembolism in both basal pyramids and the medial segment of the middle lobe (Figure 5). Despite being on postoperative anticoagulation with Clexane 0.6 mL daily, she developed pulmonary thromboembolism, leading to her readmission to the ICU for monitoring, where her Clexane dosage was increased to 0.8 mL twice daily.

Following her readmission to the ICU for pulmonary thromboembolism (PTE), the patient’s condition gradually improved under a meticulous and multidisciplinary monitoring protocol. Daily clinical assessments closely tracked respiratory function, including oxygen saturation and hemodynamic stability, while Doppler ultrasonography was used to rule out further deep vein thrombosis (DVT). CT pulmonary angiographies were performed to monitor thrombus resolution, showing gradual improvement in pulmonary circulation. Adjustments to anticoagulation therapy, including an increased dosage of Clexane to 0.8 mL twice daily, were carefully managed to ensure effective treatment while minimizing bleeding risks. Laboratory markers, such as D-dimer levels, reflected decreasing thrombotic activity over the course of treatment. As her condition stabilized, early mobilization and physiotherapy were introduced, further aiding her recovery. Within days, the patient’s respiratory symptoms resolved, and her neurological status improved significantly. She was fully conscious, cooperative, and oriented, regaining the ability to feed orally and participate in her recovery plan. This steady improvement highlighted the importance of a well-coordinated approach to managing complex postoperative complications.

**Figure 6:**
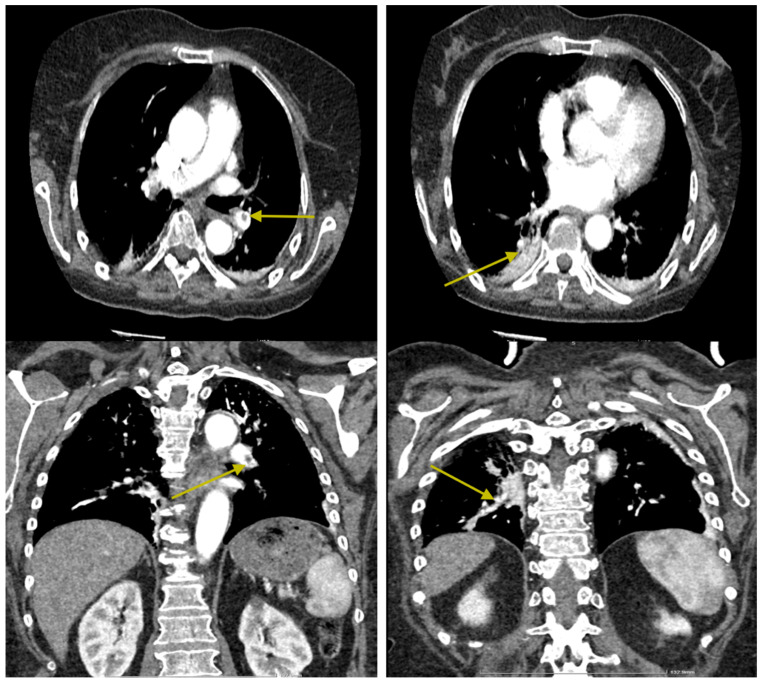
Intraluminal filling defects in the pulmonary arteries are significantly reduced in both number and size, with a few small defects persisting in the pulmonary arteries supplying the antero- and mediobasal segments (yellow arrows). Small bilateral posterior-basal atelectases are noted, with a small pseudonodular area in the right basal region showing low iodophilia, which may suggest a small pulmonary infarction. The remainder of the findings are unchanged.

The patient’s general condition improved under this treatment, and 2 days later, she was transferred back to the ward. Following a cardiology consultation, her anticoagulant therapy was switched to Xarelto 15 mg twice daily (Figure 6).

Our patient’s clinical course significantly improved; she was conscious, cooperative, and oriented in time and space, feeding orally and without a urinary catheter. One month after her admission to our clinic, she was discharged to another hospital for specialized medical recovery.

**Figure 7:**
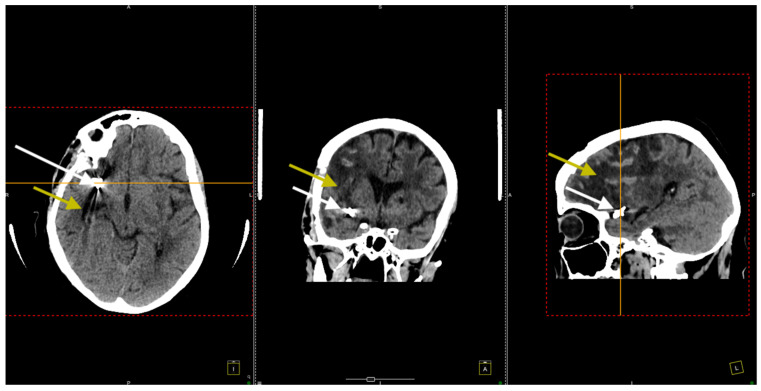
The CT scan conducted 3 months after the surgical procedure. All three sections, transversal (**left** image), coronal (**middle** image), and sagittal (**right** image), confirmed the right frontotemporal hypodensity consistent with ischemic stroke (yellow arrows) and noted artifacts from the previously placed aneurysm clips (white arrows).

At her 3-month follow-up check, a repeat native cerebral CT scan was conducted (Figure 7).

## 3. Discussion

Jefferson first described and classified “kissing” aneurysms as two aneurysms originating from distinct arteries but sharing partially adherent walls while maintaining separate origins [11].

These rare vascular anomalies account for less than 1% of all intracranial aneurysms, making their diagnosis and management particularly challenging. Their anatomical proximity often results in misdiagnosis as a single, irregular, or lobulated aneurysm, increasing the risk of suboptimal treatment and delayed intervention [12,13,14]. In this case, advanced imaging techniques, particularly 3D digital subtraction angiography (3D-DSA), were pivotal in unraveling the precise configuration of the lesions. The high-resolution visualization provided by 3D-DSA allowed the identification of dual ruptures in the M1 segment of the MCA, facilitating a carefully tailored surgical strategy. Such precise imaging underscores the critical role of technology in transforming complex cases into manageable clinical scenarios [15,16].

What makes this case particularly compelling is the convergence of two severe complications: ischemic stroke and PTE. Although kissing aneurysms frequently present with SAH due to rupture, as seen in this patient, the addition of ischemic stroke highlights the significant hemodynamic challenges and vascular compromise inherent to these lesions [14,17,18]. Microsurgical clipping was selected as the intervention of choice due to its ability to achieve immediate and definitive exclusion of both aneurysms while preserving the parent artery. This technically demanding procedure required meticulous intraoperative dissection of the shared arterial walls and the precise application of two clips to prevent residual blood flow into the lesions [19,20,21]. Table 1 illustrates that microsurgical clipping remains the most effective method for treating ruptured kissing aneurysms, particularly in complex anatomical configurations like this one. The success of this surgery reflects not only the importance of surgical expertise but also the necessity of strategic preoperative planning in cases of extreme anatomical complexity.

The postoperative development of PTE added another layer of intricacy to this patient’s care. While PTE is reported in approximately 2.3% of intracranial aneurysm surgeries, its occurrence underscores the vulnerability of postoperative patients to thromboembolic events, particularly when prolonged immobilization and hypercoagulability are present. In this case, timely diagnosis using Doppler ultrasonography and CT pulmonary angiography ensured accurate identification of deep vein thrombosis (DVT) and pulmonary artery obstruction. Anticoagulation therapy, initiated with low-molecular-weight heparin (Clexane), required careful adjustment to resolve the thromboembolic event while minimizing the risk of hemorrhagic complications [22,23]. Furthermore, early mobilization and physiotherapy were critical in reducing the likelihood of further thromboembolic episodes. This multidisciplinary approach, integrating advanced imaging, dynamic therapeutic adjustments, and proactive postoperative care, was instrumental in ensuring the patient’s favorable recovery, with no residual neurological deficits noted at the three-month follow-up.

This case not only underscores the clinical challenges of managing kissing aneurysms but also highlights the importance of addressing secondary complications such as PTE, which can significantly impact recovery. Notably, over 96.8% of reported kissing aneurysm cases are associated with favorable outcomes when managed effectively. However, the challenges posed by these lesions extend beyond their rupture risk [24,25]. As shown in Table 1, kissing aneurysms are most commonly located in the internal carotid artery, anterior communicating artery, or distal anterior cerebral arteries, with the internal carotid artery being the predominant site. Inci and Karakaya’s classification system further aids in understanding the spatial dynamics of these lesions, categorizing them as Type I (proximal/distal), Type II (superior/inferior), or Type III (right/left) configurations. The aneurysms, in this case, were classified as Type I, reflecting their proximal-distal alignment in the M1 segment of the MCA—a configuration that informed the surgical approach and clip placement [6].

While microsurgical clipping was the optimal choice in this case due to the dual ruptures and proximity of the aneurysms, alternative endovascular techniques may offer viable solutions in other scenarios. Coiling, for instance, is a minimally invasive option but poses challenges in kissing aneurysms due to the difficulty in achieving complete and simultaneous occlusion of both lesions. Stent-assisted coiling, while more effective for wide-neck aneurysms, carries the risk of hemorrhagic complications in ruptured cases due to its reliance on dual antiplatelet therapy [26,27,28]. Flow diversion, an innovative strategy for unruptured or anatomically complex aneurysms, offers the advantage of redirecting blood flow away from the aneurysm sac, promoting thrombosis while preserving the integrity of the parent vessel [21]. However, the need for prolonged antiplatelet therapy limits its applicability in ruptured cases, where immediate exclusion is critical to prevent rebleeding. As summarized in Table 1, the choice of intervention must be tailored to the patient’s clinical profile, aneurysm morphology, and rupture status.

This case illustrates the transformative potential of integrating technological advancements, surgical precision, and multidisciplinary collaboration in managing rare and challenging cerebrovascular anomalies. The dual complications of ischemic stroke and PTE serve as a reminder of the need for vigilance in both surgical planning and postoperative care. Notably, this case challenges conventional postoperative protocols, emphasizing the importance of proactive thromboembolic prevention measures, particularly in high-risk patients. Future research should focus on multicenter studies comparing microsurgical and endovascular approaches for kissing aneurysms, with an emphasis on procedural efficacy, recurrence rates, and long-term outcomes. Longer follow-up periods are essential to evaluate the durability of surgical repairs and the risk of late complications such as aneurysm recurrence or vascular restenosis. As evidenced by Table 1, variability in reported outcomes underscores the necessity for standardized data collection and reporting to refine management protocols and improve patient outcomes.

To our knowledge, this is one of the few cases in the literature detailing kissing aneurysms complicated by both ischemic stroke and PTE, making it a valuable addition to the growing body of evidence on managing multifaceted cerebrovascular conditions. By advancing our understanding of these rare anomalies, this report provides actionable insights for clinicians navigating similarly complex cases in the future.

## 4. Conclusions

This case highlights the complexities involved in diagnosing and treating kissing aneurysms of the middle cerebral artery, especially when compounded by serious complications like stroke and pulmonary thromboembolism (PTE). Despite the rarity of such cases, our patient’s successful outcome underscores the importance of accurate preoperative identification using imaging techniques like 3D digital subtraction angiography. Prompt surgical intervention through microsurgical clipping proved effective in managing the aneurysms.

The development of PTE postoperatively emphasizes the need for vigilant monitoring and early mobilization to mitigate thromboembolic risks associated with neurosurgical procedures. Adjustments in anticoagulation therapy were crucial in managing the PTE without exacerbating the risk of hemorrhagic complications, particularly given the recent intracranial surgery.

The literature indicates that while kissing aneurysms can lead to severe complications, they often have a favorable prognosis when appropriately managed. This case reinforces the necessity for a multidisciplinary approach that includes neurosurgeons, radiologists, and critical care specialists to optimize patient outcomes. Clinicians should remain alert to the potential for both neurological and thromboembolic complications, ensuring comprehensive perioperative care.

Future research should focus on developing standardized guidelines for the management of complex aneurysms and associated complications like PTE. Such efforts would contribute to improving prognoses and reducing the incidence of adverse events in patients with intricate cerebrovascular conditions.

## Figures and Tables

**Table 1:** This table presents a comprehensive literature review on cases of kissing aneurysms located in the M1 segment of the right MCA, with complications including stroke and PTE. It spans a total of 10 studies from 1978 to 2023, summarizing patient demographics, aneurysm characteristics, clinical presentations, risk factors, surgical interventions, and post-treatment complications.

Study Reference	Number of Patients	Mean Basal Age (±SD) yo	Female/Male Ratio	Mean Aneurysm Size (±SD) mm	Location of the Aneurysm in MCA	Clinical Presentation	Risk Factors	Surgical Treatment	Complication
Jefferson et al. (1978) [22]	Not specified	Not specified	Not specified	Not specified	Internal carotid artery (ICA)	Subarachnoid hemorrhage (SAH)	Not specified	Not specified	None reported
Wanifuchi et al. (2001) [19]	1	55	1:0	Not specified	Anterior communicating artery	SAH	Smoking, hypertension	Microsurgical clipping	None reported
Ide et al. (2002) [23]	1	62	Not specified	Not specified	Ophthalmic segment of ICA	SAH	Hypertension	Microsurgical clipping	None reported
Agnelli et al. (2004) [24]	Multiple	63 ± 8	0:1	5 ± 1 mm	MCA bifurcation	Ischemic stroke, SAH	Hypertension, immobilization	Endovascular surgery	Pulmonary thromboembolism
Choi et al. (2011) [25]	1	57	1:0	3 ± 1 mm	Distal MCA	SAH, stroke	Smoking, hypertension, hypercoagulability	Microsurgical clipping	None reported
Kshettry et al. (2014) [26]	Multiple	Not specified	Not specified	Not specified	Various sites	Not specified	Post-surgery hypercoagulability	Endovascular surgery	Pulmonary thromboembolism, stroke
Fu et al. (2018) [20]	1	60	0:1	Not specified	Distal anterior cerebral artery	SAH	Hypertension	Microsurgical clipping	None reported
Lee and Fang (2018) [27]	1	70	0:1	4 mm	MCA proximal segment	SAH, ischemic stroke	Recent surgery, hypertension	Microsurgical clipping	Pulmonary thromboembolism
Inci and Karakaya (2021) [6]	30	58 ± 10	Not specified	6 ± 2 mm	Various sites in MCA, ICA	SAH	Hypertension, smoking	Microsurgical clipping, endovascular	Stroke, rebleeding
Pan et al. (2023) [28]	Multiple	55 ± 12	Not specified	Not specified	Various sites, including MCA	Not reported	Surgery, immobilization	Endovascular surgery	Pulmonary thromboembolism (2.3%)

## Data Availability

The data presented in this study are available on request from the corresponding author.

## References

[1] Hanel R.A., Spetzler R.F. (2008). Surgical treatment of complex intracranial aneurysms. Neurosurgery.

[2] Ravina K., Rennert R.C., Kim P.E., Strickland B.A., Chun A., Russin J.J. (2019). Orphaned Middle Cerebral Artery Side-to-Side In Situ Bypass as a Favorable Alternative Approach for Complex Middle Cerebral Artery Aneurysm Treatment: A Case Series. World Neurosurg..

[3] Etminan N., Brown R.D., Beseoglu K., Juvela S., Raymond J., Morita A., Torner J.C., Derdeyn C.P., Raabe A., Mocco J. (2015). The unruptured intracranial aneurysm treatment score. Neurology.

[4] Shao M.M., White T.G., Bassett J.B., Dowlati E., Mehta S.H., Werner C., Golub D., Shah K.A., Dehdashti A.R., Teron I. (2024). Intrasaccular Treatment of Intracranial Aneurysms: A Comprehensive Review. J. Clin. Med..

[5] Lu Y., Ding C., Tan S., Zhou X., Wang Y. (2023). Predisposing factors for the deformation of parent artery of anterior circulation saccular aneurysm after stent-assisted embolization: A retrospective cohort study. Interv. Neuroradiol..

[6] Inci S., Karakaya D. (2021). Kissing Aneurysms: Radiological and Surgical Difficulties in 30 Operated Cases and a Proposed Classification. World Neurosurg..

[7] Wessels L., Fekonja L.S., Achberger J., Dengler J., Czabanka M., Hecht N., Schneider U., Tkatschenko D., Schebesch K.-M., Schmidt N.O. (2020). Diagnostic reliability of the Berlin classification for complex MCA aneurysms—usability in a series of only giant aneurysms. Acta Neurochir..

[8] Cockroft K.M., Marks M.P., Steinberg G.K. (2000). Planned Direct Dual-modality Treatment of Complex Broad-necked Intracranial Aneurysms: Four Technical Case Reports. Neurosurgery.

[9] O’Neal C.M., Ernst G.L., Hughes K.L., Stephens T.M., Hendrix M.C., Gross N.L., Bohnstedt B.N., Cheema A.A. (2021). Reported incidence and treatment modalities of giant cerebral aneurysms in the pediatric population: A systematic review and illustrative case report. J. Clin. Neurosci..

[10] Lu X., Huang Y., Zhou P., Zhu W., Wang Z., Chen G. (2021). Cerebral revascularization for the management of complex middle cerebral artery aneurysm: A case series. Exp. Ther. Med..

[11] Singh D.K., Sharma P.K., Singh A.K., Chand V.K. (2023). Ruptured mirror DACA aneurysm: A rare case report and review of literature. J. Cerebrovasc. Endovasc. Neurosurg..

[12] Ryška P., Lojík M., Habalová J., Kajzrová C., Česák T., Vítková E., Bartoš M., Bělobrádek Z., Krajina A. (2024). Endovascular Therapy of Ruptured Aneurysms on Moyamoya Collateral Vessels: Two Cases. Medicina.

[13] Zhou Z., Lan W., Yu J. (2023). Endovascular treatment of middle cerebral artery aneurysms: Current status and future prospects. Front. Neurol..

[14] Magid-Bernstein J., Girard R., Polster S., Srinath A., Romanos S., Awad I.A., Sansing L.H. (2022). Cerebral Hemorrhage: Pathophysiology, Treatment, and Future Directions. Circ. Res..

[15] Dobrocky T., Matzinger M., Piechowiak E.I., Kaesmacher J., Pilgram-Pastor S., Goldberg J., Bervini D., Klail T., Pereira V.M., Z’Graggen W. (2023). Benefit of Advanced 3D DSA and MRI/CT Fusion in Neurovascular Pathology. Clin. Neuroradiol..

[16] Guo J., Li C., Yu P., Xu T., Zhou H., Chen H. (2023). The effect of low molecular weight heparin combined with air pressure in the prevention of lower extremity venous thrombosis after cesarean section: A single-center retrospective study. Medicine.

[17] Németh T., Hausinger P., Márkos-Gergely G., Gyura E., Wasserberg J., Barzó P. (2024). Exclusion of the anterior communicating artery with endovascular flow diverters – A possible treatment method of a wide-necked aneurysm. Interdiscip. Neurosurg..

[18] Mosteiro A., Pedrosa L., Codes M., Reyes L., Werner M., Amaro S., Enseñat J., Rodríguez-Hernández A., Aalbers M., Boogaarts J. (2024). Microsurgical and endovascular treatment of large and giant aneurysms of the anterior circulation: A systematic review. Brain Spine.

[19] Wanifuchi H., Shimizu T., Higa T., Nakaya K. (2001). Kissing Mirror Image Anterior Communicating Artery Aneurysms. Neurol. Med. Chir..

[20] Fu C.-Y., Chen J.-L., Liu Z.-H., Wang P.-C., Duan C.-Z., Zhao J.-N. (2018). Kissing aneurysms of the distal anterior cerebral artery: A case report and literature review. Exp. Ther. Med..

[21] Solis F., Plasencia A., Wahlster S., Walker M., Levitt M.R., Ecos R. (2023). Flow Diversion for the Treatment of Intracranial Aneurysms in a Peruvian Cohort: Experiences from a Limited-Resource Setting and Barriers to Implementation. World Neurosurg..

[22] Jefferson A. (1978). The significance for diagnosis and for surgical technique of multiple aneurysms of the same internal carotid artery. Acta Neurochir..

[23] Ide M., Hagiwara S., Tanaka N., Kawamura H. (2002). Bilateral Ophthalmic Segment “Kissing” Aneurysms Presenting With Subarachnoid Hemorrhage. Neurol. Med. Chir..

[24] Agnelli G. (2004). Prevention of Venous Thromboembolism in Surgical Patients. Circulation.

[25] Choi C.-Y., Han S.-R., Yee G.-T., Lee C.-H. (2011). Kissing aneurysms of the distal anterior cerebral artery. J. Clin. Neurosci..

[26] Kshettry V.R., Rosenbaum B.P., Seicean A., Kelly M.L., Schiltz N.K., Weil R.J. (2014). Incidence and risk factors associated with in-hospital venous thromboembolism after aneurysmal subarachnoid hemorrhage. J. Clin. Neurosci..

[27] Lee W.-C., Fang H.-Y. (2018). Management of pulmonary embolism after recent intracranial hemorrhage: A case report. Medicine.

[28] Pan J., Bonow R.H., Temkin N., Robinson E.F., Sekhar L.N., Levitt M.R., Lele A.V. (2023). Incidence and Risk Model of Venous Thromboembolism in Patients with Aneurysmal Subarachnoid Hemorrhage. World Neurosurg..

